# The Clinical Features and Prognostic Factors for Treatment Outcomes of Dematiaceous Fungal Keratitis over 9 Years at a Tertiary Eye Care in Northern Thailand

**DOI:** 10.3390/jof7070526

**Published:** 2021-06-30

**Authors:** Chulaluck Tangmonkongvoragul, Susama Chokesuwattanaskul, Napaporn Tananuvat, Monsicha Pongpom, Phit Upaphong, Sinthirath Saysithidej, Muanploy Niparugs, Siriporn Chongkae

**Affiliations:** 1 Department of Ophthalmology, Faculty of Medicine, Chiang Mai University, Chiang Mai 50200, Thailand; dr_susama_c@yahoo.com (S.C.); napaporn.t@cmu.ac.th (N.T.); peachapn@hotmail.com (P.U.); ployname@yahoo.com (M.N.); 2 Department of Microbiology, Faculty of Medicine, Chiang Mai University, Chiang Mai 50200, Thailand; monsicha.p@cmu.ac.th (M.P.); siripornannt4@gmail.com (S.C.); 3 National Ophthalmology Center, Vientiane 0100, Laos; sinsay110@hotmail.com

**Keywords:** dematiaceous fungi, fungal keratitis, PCR, prognostic factor

## Abstract

Dematiaceous fungal keratitis is an important etiology of visual loss, particularly in an agricultural society. From a retrospective review of medical records from 2012 to 2020, 50 keratitis cases of cultured-positive for dematiaceous fungi were presented at a tertiary care hospital in Northern Thailand. The study aimed to identify the isolated causative dematiaceous species using the PCR technique and to explore their related clinical features, including treatment prognoses. Sequencing of the amplified D1/D2 domains and/or ITS region were applied and sequenced. Of the 50 dematiaceous fungal keratitis cases, 41 patients were males (82%). In most cases, the onset happened during the monsoon season (June to September) (48%). The majority of the patients (72%) had a history of ocular trauma from an organic foreign body. The most common species identified were *Lasiodiplodia* spp. (19.35%), followed by *Cladosporium* spp. and *Curvularia* spp. (12.90% each). About half of the patients (52%) were in the medical failure group where surgical intervention was required. In summary, ocular trauma from an organic foreign body was the major risk factor of dematiaceous fungal keratitis in Northern Thailand. The brown pigmentation could be observed in only 26%. Significant prognostic factors for medical failure were visual acuity at presentation, area of infiltrate, depth of the lesions, and hypopyon.

## 1. Introduction

Fungal keratitis remains one of the significant ophthalmologic conditions leading to visual impairments in an agricultural society [1,2]. Typically, clinical symptoms include subacute painful visual loss with redness, excessive tearing or discharge, and photophobia. In a severe case, fungal keratitis can progress to endophthalmitis [3]. The most common fungal pathogens include *Aspergillus* spp., *Fusarium* spp., and dematiaceous fungi. 

In previous studies, the proportion of dematiaceous fungi as a causative organism of fungal keratitis varied from 4.4 to 44.2% [1,4,5,6,7,8,9]. Dematiaceous fungi are ubiquitously found in soil, therefore, any agricultural-related accidents can introduce the fungi from the vegetative materials into the eyes, resulting in keratitis. Consequently, dematiaceous fungal keratitis is considered a major infectious ophthalmic etiology of visual loss, particularly in an agricultural setting [10]. Although the typical clinical characteristics of dematiaceous fungal keratitis can be very distinctive, with dark (either black or brown) pigments stained on the ulcer, the non-pigmented lesions are not uncommon. Moreover, the fungi generally penetrate deep into the cornea, so with conventional corneal scraping and potassium hydroxide (KOH) stain, the yield of fungus is usually inadequate to confirm the diagnosis. Using calcofluor white combined with potassium hydroxide can improve the diagnostic sensitivity up to 98.3%. Additionally, the polymerase chain reaction (PCR) technique has been shown to further enhance the sensitivity and specificity over smear stains and culture [11,12]. With limited accessibility to PCR methodology for definitive microbiological identification in a routine laboratory, the clinical characteristics and visual outcomes of dematiaceous fungal keratitis remain obscured. 

There have been a limited number of studies on dematiaceous fungal keratitis in Thailand, partly due to the lack of definitive microbiological results for the identification of the species [8,13]. However, as most dematiaceous fungal keratitis cases are severe and often require corneal transplantation, the specimens are available for further microbiological identification. Though a great variety of dematiaceous fungi have been reported to cause keratitis in humans, the differences in clinical characteristics and visual prognoses have been rarely studied due to the limited number of cases. This study is the largest case series of dematiaceous fungal keratitis in Thailand, including the clinical characteristics, fungal identification using PCR technique, risk factors, disease progression, and treatment prognoses in some species of dematiaceous fungi. 

## 2. Materials and Methods

This retrospective descriptive study was conducted in accordance with the tenets of the Declaration of Helsinki and the protocol was approved by the Ethics Committee of the Faculty of Medicine, Chiang Mai University. The medical records of patients diagnosed with dematiaceous fungal keratitis from 2012 to 2020 were retrospectively reviewed. Demographic data, baseline clinical characteristics, treatments, and visual outcomes were included. The anterior photographs of the patients with dematiaceous fungal keratitis were re-evaluated by a single corneal specialist (C.T.) to accurately describe the characteristics of the lesions.

All patients underwent a thorough slit-lamp examination and anterior segment photography with a routine workup for various microorganisms using standard techniques. Corneal scrapings were performed under slit-lamp biomicroscope by ophthalmologists after topical anesthesia (Tetracaine hydrochloride 0.5%) instillation. Each scraping was processed using Gram stain, potassium hydroxide 10% (KOH) wet mount, and calcoflour white-KOH staining (CWS). The specimens were also directly inoculated in Sabouraud dextrose agar stored at room temperature. Fungal identification was based on the macroscopic and microscopic appearances. Fungal classification could be refined upon the production and accumulation of the melanin pigment within the cell wall. Dematiaceous fungi accumulate melanin pigment within walls revealing a brown to black hyphal color. Therefore, an isolate showing a brown hyphal color and dark colony color at both the surface and the reverse side is considered as dematiaceous fungus.

### 2.1. Dematiaceous Fungal Identification Using Polymerase Chain Reaction (PCR) Technique and Sequencing Analysis

Dematiaceous fungus obtained from the patients’ corneal tissues were cultured. Then, the dematiaceous fungal DNA was extracted from the colonies for further identification [14].

### 2.2. DNA Extraction

Approximately 0.5 g of fungus were suspended in 600 µL extraction buffer (10 mM Tris, 1 mM EDTA, 1% *w*/*v* sodium dodecyl sulphate); 0.5 g of 0.5 mm sterile glass beads was then added. The fungal hyphae were mechanically broken in a SpeedMill Plus bead beater (Analytik Jena AG Inc., Jena, Germany). The lysate was centrifuged to precipitate the glass beads and cell debris at 13,000× *g* for 10 min in a microcentrifuge. Supernatants were DNA extracted with an equal volume of phenol-chloroform-isoamyl alcohol (25:24:1 *v*/*v*, Usb Corporation, Cleveland, OH, USA). The sample DNA was precipitated with 3 M sodium acetate and cold absolute ethanol (−20 °C), dried, and resuspended in 50 µL distilled water.

### 2.3. Amplification and Sequencing of D1/D2 Domains of the Large Subunit Ribosomal DNA (Lsurdna) and Internal Transcribed Spacer (ITS) Region of the Ribosomal rDNA

Primers NL-1 (5′-GCATATCAATAAGCGGAGGAAAAG-3′) and NL-4 (5′-GGTCCGTGTTTCAAGACG-3′) and ITS primers ITS-1 (5′-TCCGTAGGTGAACCTGCGG -3′) and ITS-4 (5′-TCCTCCGCTTATTGATATGC-3′) were used for the amplification of the D1/D2 domains of the LSUrDNA and the internal transcribed spacer (ITS) region of the ribosomal DNA. The polymerase chain reaction (PCR) containing 100 ng of template DNA, 0.5 µM of each primer, 200 µM each dNTP, and, and 1 U of Phusion DNA polymerase (New England Biolabs Inc., Ipswich, MA, USA) were prepared. Amplification was performed with a T100 PCR Thermal Cycler (Bio-Rad Laboratories, Inc. Hercules, CA, USA) under the following conditions: 1 cycle of 98 °C for 30 s followed by 35 cycles of 98 °C for 10 s (denaturation step), 55 °C for 30 s (annealing step), and 72 °C for 30 s (extension step), with a final extension at 72 °C for 10 min. The amplified DNA products were purified with a GeneJET gel extraction kit (Thermo Scientific, Vilnius, Lithuania) and sent for sequencing at ATCG Company Limited (Thailand Science Park (TSP)). The sequencing was performed by labeling with BigDye terminator (Applied Biosystems, Foster City, CA, USA) and the labeled DNA was subjected to sequencing with an ABI3730XL sequencer (Applied Biosystems).

The primer pairs, NL-1/NL-4 and ITS1/ITS4 were used to sequence each amplicon. The obtained data were used to search for sequence identity by using a nucleotide BLAST (BlastN) on the National Center for Biotechnology Information (NCBI) database (www.ncbi.nlm.nih.gov/BLASTN; accessed during September 2020 to February 2021). Identification of the dematiaceous fungi was made based on one hundred percent matching or the highest score to the subjected DNA sequence.

### 2.4. Statistical Analysis

Descriptive statistics were used to describe the demographic data, baseline clinical characteristics, treatments, and visual outcomes. Microsoft Excel XP and R statistics (version 3.4.4, R-project (2018), Austria) were used to perform the statistical analysis.

## 3. Results

From a retrospective review of medical records from 2012 to 2020, 50 keratitis cases of cultured-positive for dematiaceous fungi have presented at Chiang Mai University Hospital. This hospital is located in Chiang Mai province, which is 685 km (426 mi) north of Bangkok. Chiang Mai has a tropical wet and dry climate, tempered by the low latitude and moderate elevation, with warm to hot weather year-round, though nighttime conditions during the dry season can be cool and are much lower than daytime highs. 

Of those 50 cases, dematiaceous fungi could be identified by conventional method (KOH examination/calcofluor white-KOH staining) in 30 cases. Most of them were unidentified species. For further definitive microbiological identification, using PCR and sequencing technology were required. After receiving a grant for this study from the Faculty of medicine in the year 2020, the fungal cultures from corneal specimens of only 22 cases were available for further species identification using the PCR technique. The incidence, including the clinical characteristics, risk factors for determining the final visual prognosis, and treatment outcomes, were analyzed.

### 3.1. Epidemiological Features

**Figure 1:**
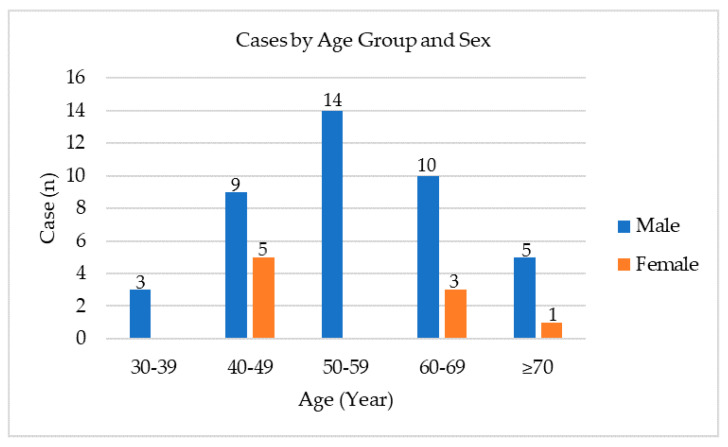
Distribution of dematiaceous fungal keratitis cases by age group and sex.

Of 50 patients diagnosed with dematiaceous fungal keratitis, most of the patients were males (41/50, 82%). The mean (±SD) age at presentation was 55 (±11) years. On most occasions, the onset happened during monsoon season in Thailand (Jun to Sep) (24/50, 48% of the patients). The mean (±SD) visual acuity at presentation was 1.80 (±0.98) logMAR units (Snellen 6/360). Nearly a quarter (11/50, 22%) of the patients had topical steroids at the time of diagnosis, either from underlying ophthalmic conditions, self-medication, or initial treatment. The mean (±SD) time to initial presentation was 11 (±12) days, and only 30% (15/50) of patients had an initial diagnosis of presumed fungal keratitis by clinical diagnosis. Because lacking antifungal eye drops in some rural hospitals, only 20% (10/50) of patients received the initiation of antifungal therapy from primary hospitals (Table 1 and Figure 1).

Most of the patients (37/50, 74%) had a history of foreign body exposure. As high as 97% (36/37) of the foreign bodies were organic, particularly plants (8/37, 22%), wood (6/37, 16%), and contaminated soil (4/37, 11%). Only 30% of the patients had systemic risk factors of either diabetes mellitus (DM) or human immunodeficiency virus (HIV) infection (Table 2).

### 3.2. Clinical Characteristics

**Figure 2:**
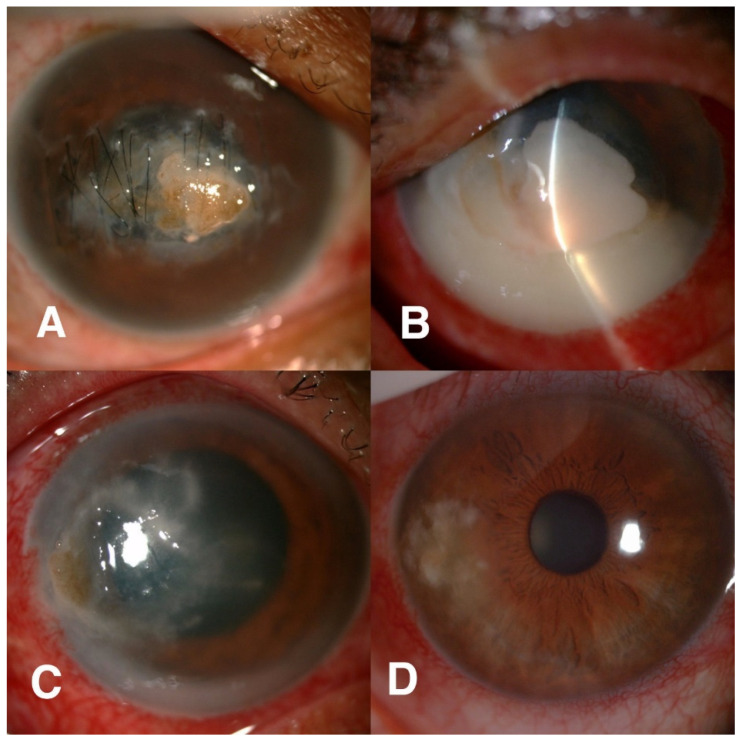
Clinical photograph of dematiaceous keratitis cases. (**A**) Clinical photograph showing diffuse brown pigmentation in the anterior stroma; (**B**) clinical photograph under silt-beam illumination showing a pigmented raised/elevated lesion; (**C**) clinical photograph showing a ring infiltrate and feathery borders; (**D**) clinical photograph showing a dendritic lesion in the inferotemporal quadrant of cornea.

Only 13 of 50 (26%) patients had brown pigmentation on ulcers. Other clinical characteristics included wet appearance (27/50, 54%), pigmented epithelial plaque (6/50, 12%), feathery edge (39/50, 78%), ring infiltrate (12/50, 24%), endothelial plaque (16/50, 32%), and hypopyon (33/50, 66%). Of 50 eyes, six patients (6/50, 12%) had corneal perforations during the treatments. (Table 3 and Figure 2).

### 3.3. Laboratory Finding

**Figure 3:**
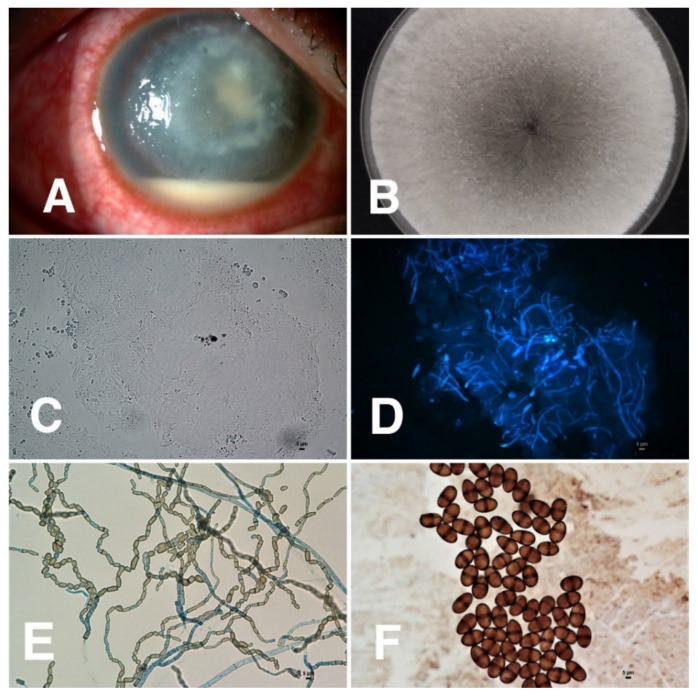
Clinical characteristics of *Lasiodiplodia* keratitis and microbiological identification from corneal scraping specimens. (**A**) *Lasiodiplodia* keratitis showing a feathery edge, hypopyon, and macroscopic pigmentation; (**B**) cultivation on Sabouraud’s dextrose agar at 28 °C for 3 and 7 days shows white fluffy colony that becomes jet black when aged; (**C**) 10% KOH mount showing fungal hyphae, magnification 400; (**D**) fluorescent microscopic picture was taken after calcofluor white staining, magnification 400; (**E**,**F**) microscopic examination of the slide culture shows brown septate hyphae at 400 magnification and two-celled arthroconidia at 1000 magnification.

**Figure 4:**
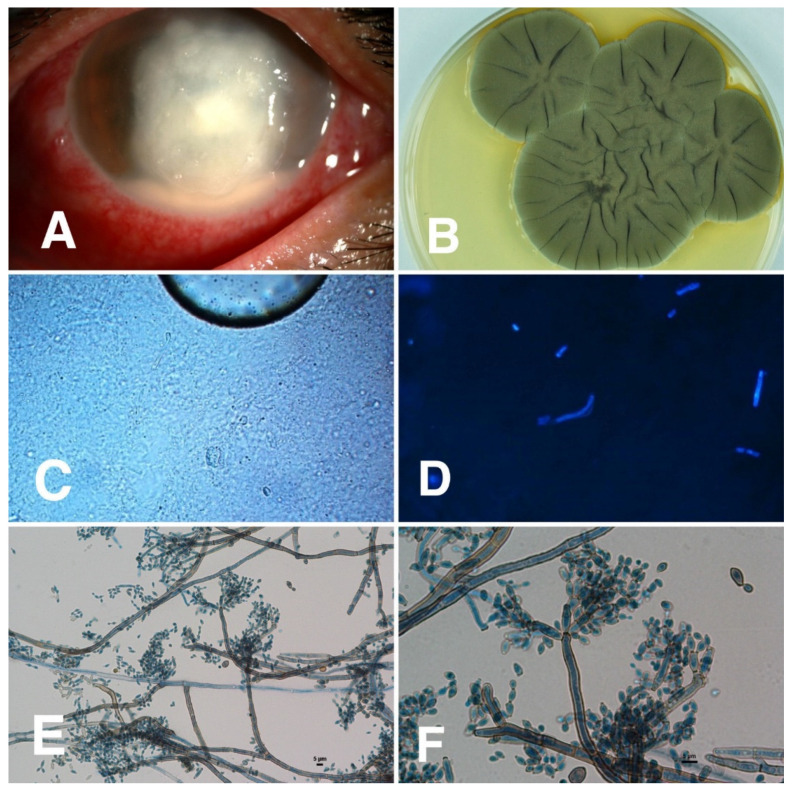
Clinical characteristics of *Cladosporium* keratitis and microbiological identification from corneal scraping specimens. (**A**) *Cladosporium* keratitis showing both feathery edge and hypopyon; (**B**) cultivation on Sabouraud’s dextrose agar at 28 °C shows grayish-green velvety colony; (**C**) 10% KOH mount showing fungal hyphae, magnification 400; (**D**) fluorescent microscopic picture was taken after calcofluor white staining, magnification 400; (**E**,**F**) microscopic examination of the slide culture showing conidia with scars, at 100 and 400 magnifications.

**Figure 5:**
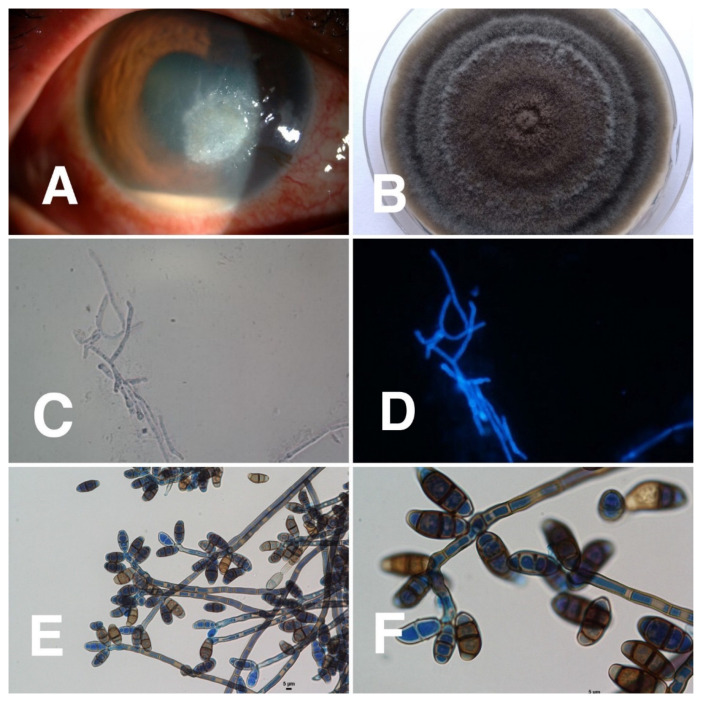
Clinical characteristics of *Curvularia keratitis* and microbiological identification from corneal scraping specimens. (**A**) *Curvularia keratitis* showing a raised lesion, feathery edge, and macroscopic central pigmentation; (**B**) cultivation on Sabouraud’s dextrose agar at 28 °C shows black velvety colony; (**C**) 10% KOH mount showing fungal hyphae, magnification 400; (**D**) fluorescent microscopic picture was taken after calcofluor white staining, magnification 400; (**E**,**F**) microscopic examination of the slide culture shows curved macroconidia with central enlargement, at 100 and 400 magnifications.

**Figure 6:**
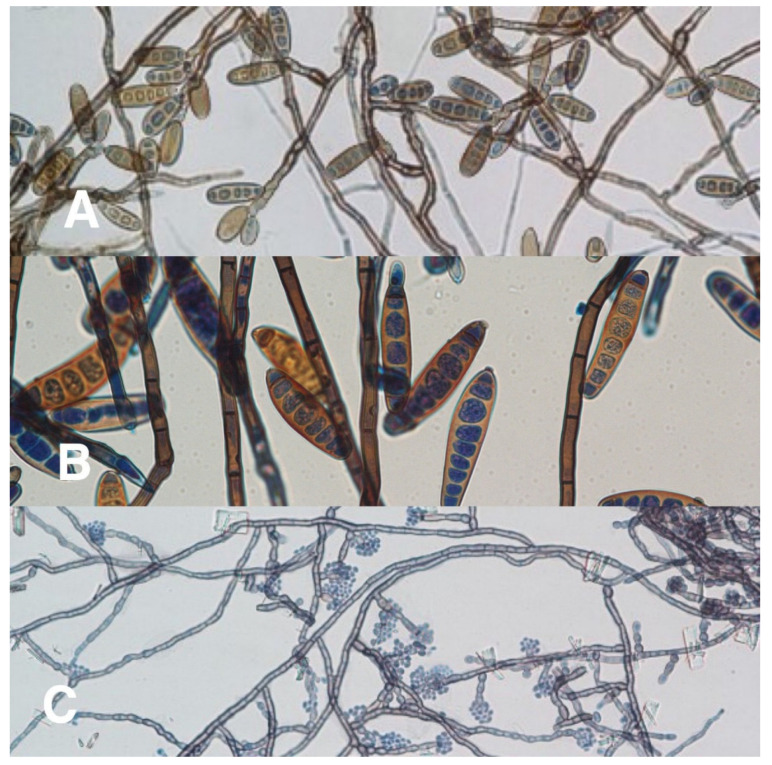
Microbiological identification of dematiaceous keratitis from corneal scraping specimens at 400 magnification. (**A**) Microscopic examination of *Bipolaris* spp. obtained from a slide culture; (**B**) microscopic examination of *Exerohilum* spp. obtained from a slide culture; (**C**) microscopic examination of *Phialophora* spp. obtained from slide culture.

Of 50 corneal specimens of patients with confirmed diagnoses of dematiaceous fungal keratitis, the species were identified in 31 specimens by either PCR or fungal morphological-based study. The most common species identified were *Lasiodiplodia* spp. (6/31, 19%), followed by *Cladosporium* spp. and *Curvularia* spp. (4/31, 13% each). *Bipolaris* spp., *Colletotrichum* spp., *Exserohilum* spp., and *Phialophora* spp. were also found. (2/31, 6% each) (Table 4 and Figure 3, Figure 4, Figure 5 and Figure 6). Sequenced data for species identification was in the Supplementary result.

### 3.4. Final Treatment Outcomes

Topical natamycin 5% (26/50) and ketoconazole 2% (19/50) were preferred medications. Oral itraconazole was prescribed in all patients. Subconjunctival fluconazole (21/50) and intracameral amphotericin B (2/50) were adjunctive therapy in severe cases. The final outcomes of the treatments were classified as either medication success or medication failure. The patients in the medication success group required no surgical intervention during the treatments until the final follow-up. Approximately half of the patients (24/50, 48%) were identified as medication success (Table 5).

In the univariate analysis, the predicting factors for medication failure included visual acuity at presentation (*p* = 0.002), area of infiltrate (*p* = 0.002), depth of the lesions (*p* = 0.007), hypopyon (*p* = 001), and location of ulcer (*p* = 0.002) (Table 6)

### 3.5. Clinical Characteristics and Treatment Outcomes of Top 3 Most Common Dematiaceous Fungi Causing Keratitis

In the subgroup analysis of the three most common dematiaceous species, the mean (±SD) presenting VA of *Cladosporium* spp., *Curvularia* spp., and *Lasiodiplodia* spp. were 2.43 (±0.44), 1.55 (±1.17), and 2.01 (±0.69) LogMAR units, respectively. Most of the cases (50–75%) had been exposed to organic foreign bodies. For clinical characteristics, 25% (1/4) of the lesions from *Curvularia* spp. and *Lasiodiplodia* spp. were pigmented, but none (0/4, 0%) of the lesions from *Cladosporium* spp. (Table 7).

## 4. Discussion

This study provides etiology, including the clinical characteristics and determining factors for the treatment outcomes of dematiaceous fungal keratitis in Northern Thailand. From our results, most of the dematiaceous fungal keratitis patients were males, aged 50–59 years, and presented to the hospital during monsoon season. As many as 74% of the patients had a history of agricultural-related foreign body exposure, including plants, wood, and contaminated soil. Consistent with our findings, dematiaceous fungi are primarily found in soil or associated with plants, especially in tropical and subtropical regions [4,15]. Significant risk factors were also corneal trauma from organic materials and male gender [15]. Since Thailand is mainly an agricultural society, during the monsoon season (June to September), most Thai people are exposed to soil and plants while planting and farming. This finding emphasizes the roles of public education and prevention programs to help reduce the numbers of organic ocular trauma associated with fungal keratitis.

Basically, the morphological identification of dematiaceous fungi is based on the presence of dark vegetative hyphae due to melanin accumulation in their cell wall, so-called pigmented fungus [16]. The pigments are expected to be clinically present on any lesions caused by the dematiaceous fungi, however, in our study, the characteristics of pigmentation in corneal ulcer were observed in only one-quarter of the patients, in which all were brown. From previous reports, the characteristic macroscopic pigmentation varied from 14.5–66.7% of dematiaceous fungal keratitis cases [6,17,18]. The lack of pigment in some dematiaceous fungal keratitis patients might occur from the intense inflammation that obscured the gross pigmentation [19]. In our results, only 30% of patients were accurately diagnosed with fungal keratitis from their primary hospitals. Even when corneal scraping was performed by a corneal specialist, the fungal culture only had a sensitivity of 73%, therefore, misdiagnosis is not uncommon [20].

The data on the incidence of fungal keratitis from Northern Thailand are limited. According to two retrospective studies, the incidence of fungal keratitis was 46.3% of all microbial keratitis cases [8]. *Fusarium* spp., dematiaceous fungi, and *Aspergillus* spp. were the top three most common pathogens [13]. This agrees with studies in India and Nepal in which the dematiaceous fungi has also been reported to be the second most common fungal pathogen [6,21,22]. With increasing evidence, the trends of dematiaceous fungal keratitis are increased in tropical and subtropical climate locations. Previous studies have indicated that the most frequently associated dematiaceous species were *Curvularia* spp., *Alternaria* spp., *Exserohilum* spp., and *Cladosporium* spp. [22,23,24,25]. Among these species, *Curvularia* spp. was the most common cause of keratitis [4,6,15,25,26]. However, in our study, *Lasiodiplodia* spp. was the most common species, followed by *Curvularia* spp. and *Cladosporium* spp.

*Lasiodiplodia* spp. was the most common species causing dematiaceous fungal keratitis in our study. The finding is striking as the reported incidence of *Lasiodiplodia* keratitis is about 0.5–9% of dematiaceous fungal keratitis cases and only 47 cases have been published so far, mostly in India, Sri Lanka, and the Philippines [4,27,28,29,30]. There have been only two cases of *Lasiodiplodia* keratitis reported in Thailand since 2015 [31]. *Lasiodiplodia* spp. is a common plant pathogen in tropical and subtropical countries. Infections in humans are rare and most frequently involve the cornea. Most of our cases had ocular trauma with contaminated soil, which is similar to previous studies [27,29]. The predominant clinical characteristics of our cases were consistent with typical features of other fungal keratitis cases, including feathery edge (83%) and hypopyon (67%). Compared to the other dematiaceous species reported in our study, most cases of *Lasiodiplodia* keratitis were in the medical failure group. Five of six cases underwent penetrating keratoplasty which might be explained by the larger area of infiltrate at presentation relative to the other two common species. There is limited data regarding the visual outcomes of this unique dematiaceous species. However, Lekhanon et al. also reported that *Lasiodiplodia* keratitis caused a very severe type of keratitis, and penetrating keratoplasty was the beneficial treatment [31].

Among the three most common dematiaceous species reported in our study, *Cladosporium* spp. demonstrated the most severe clinical presentation in terms of poor presenting visual acuity, and the worst treatment outcomes, in which half of the patients required enucleation/evisceration. Emphatically, 2 of 6 eviscerated/enucleated patients in our case series were infected with *Cladosporium* spp. Of note, the common clinical characteristics of *Cladosporium* spp. were feathery edge (25%) and hypopyon (100%), with no pigment, making it indistinguishable from other microorganisms and thus likely to be misdiagnosed, causing delays in the antifungal treatment. Moreover, all *Cladosporium* keratitis cases had hypopyon, which may imply that hypopyon is related to the high rate of medication failure for this specific species. 

*Curvularia* spp. was reported as the most common dematiaceous fungi causing keratitis in India and the USA [15,26]. About 50–69% of cases had a history of trauma related to organic materials, similar to our results (75%) [26,32]. In general, clinical features of *Curvularia keratitis* in our study shared the common clinical signs with other fungal keratitis lesions including raised (75%), epithelial plaque (50%), feathery edge (50%), and hypopyon (16.5%), but with pigmentation in only 25% of the cases. In this group, patients were presented to the hospital earlier (approximately 4 days) than the other two common species, *Cladosporium* spp. and *Lasiodiplodia* spp. (approximately 2–3 weeks). Moreover, around 25% of cases had a correct primary diagnosis of fungal keratitis. In 2020, Khurana et al. reported that the most common characteristics of *Curvularia keratitis* were feathery edge (73%), followed by raised or elevated lesion (53%) [32]. Previous studies revealed the distinguishing characteristics of raised lesions in dematiaceous fungal keratitis, compared to feathery edges and hypopyon that were commonly found in both dematiaceous and hyaline fungal keratitis [33]. It is likely to presume that a raised or elevated lesion is a pathognomonic sign of *Curvularia keratitis*. For the treatment outcomes, half of our cases underwent penetrating keratoplasty compared to 5–19% from previous reports [26,32].

For subgroup analysis, predicting factors for medication failure in this study, including poor presenting visual acuity, a large area of infiltrate, the presence of hypopyon, and full-thickness lesions, may prompt aggressive treatments at the beginning, as patients were likely to undergo surgical interventions. Interestingly, higher proportions of the patients in the medication success group had the primary ulcer located in the central cornea compared to the paracentral and total cornea in the medication failure group. This may be explained by substantial visual obscuration of central corneal involvement relative to the paracentral or limbal area, thus prompting earlier medical visits.

Due to the retrospective study, some clinical history and characteristics were not present in the medical records. The clinical characteristics were mainly evaluated from the anterior photographs of the cornea by the corneal specialist (C.T.) to accurately describe the lesions. As the molecular-based technique required the colonies of dematiaceous fungi from the corneal specimens, only 22/50 (44%) of the samples were available at the time of the study. A further prospective study in multi-centers could provide more dematiaceous fungal keratitis specimens. 

## 5. Conclusions

The increasing trends of fungal keratitis caused by dematiaceous fungi are a new challenge in Thailand and other Asian countries. In patients with dematiaceous fungal keratitis, not all the patients contained the classic clinical characteristics of fungal keratitis. The brown pigmentation could be observed in only 13/50 (26%) patients. *Cladosporium* spp. provided the worst clinical outcomes, in which half of the patients required enucleation/evisceration. A raised or elevated lesion was a possible pathognomonic sign of *Curvularia keratitis*. *Lasiodiplodia* spp. was the most common species causing dematiaceous fungal keratitis in this study, in which feathery edge (83%) and hypopyon (67%) were the predominant signs. The predicting factors for medication failure included poor presenting visual acuity, a large area of infiltrate, depth of the lesions, and hypopyon.

## Figures and Tables

**Table 1:** Demographics and baseline clinical characteristics of patients with dematiaceous fungal keratitis (*N* = 50).

Demographics and Baseline Clinical Characteristics	Cases
Age (mean, SD)	55, 11
Sex (male: female)	41:9
Season at onset (n, %)	
- Monsoon (Jun to Sep)	24/50, 48
- Winter (Oct to Jan)	17/50, 34
- Summer (Feb to May)	9/50, 18
Underlying ocular disease (n, %)	11/50, 22
- Previous globe rupture (n, %)	3/50, 6
Previous use of topical steroid (n, %)	11/50, 22
Time to the presentation (days) (mean, SD)	11, 12
Visual acuity at presentation (LogMAR) (mean, SD)	1.80, 0.98
Primary diagnosis as fungal keratitis (n, %)	15/50, 30
Initiation of antifungal therapy from the primary hospital (n, %)	10/50, 20

Abbreviations: SD = standard deviation, LogMAR = the Logarithm of the minimum angle of resolution. (LogMAR of 0 indicated a Snellen score of 6/6 and LogMAR of 1 indicated a Snellen score of 6/60).

**Table 2:** Predisposing factors in patients with dematiaceous fungal keratitis (*N* = 50).

Predisposing Factors	Cases (%)
Ocular	
Trauma	
- Foreign body exposure	
- Organic foreign body	36 (72%)
- Non-organic foreign body	1 (2%)
- Penetrating globe injury	3 (6%)
Non-Trauma	
- Previous surgery	8 (16%)
- Topical steroid use	11 (22%)
**Systemic**	
- Diabetes mellitus	7 (14%)
- HIV infection	1 (2%)

Abbreviation: HIV = human immunodeficiency virus.

**Table 3:** Clinical characteristics of dematiaceous fungal keratitis (*N* = 50).

Clinical Characteristics of the Lesion	Cases (%)
*Area of infiltrate (mm^2^) (mean, SD)*	27.61, 34.05
*Location (N, %)*	
- Paracentral	21 (42)
- Central	17 (34)
- Total	8 (16)
- Limbus	4 (8)
*Color (N, %)*	
- White	25 (50)
- Yellow	25 (50)
*Pigmentation (N, %)*	
- None	37 (74)
- Brown	13 (26)
- Black	0 (0)
*The appearance of the lesion (N, %)*	
- Wet	27 (54)
- Dry	23 (46)
*Raised/Elevated (N, %)*	21 (42)
*Epithelial plaque (N, %)*	
- None	31 (62)
- Raised/Elevated	13 (26)
- Pigmentation	6 (12)
*Feathery edge (N, %)*	39 (78)
*Ring infiltrate (N, %)*	12 (24)
*Dendritic (N, %)*	17 (34)
*Satellite (N, %)*	12 (24)
Depth (*N*, %)	
- Full corneal thickness	26 (52)
- Anterior two-thirds of corneal thickness	19 (38)
- Anterior one-third of corneal thickness	5 (10)
*Endothelial plaque (N, %)*	16 (32)
*Perforation (N, %)*	6 (12)
*Endophthalmitis (N, %)*	2 (4)
*Hypopyon (N, %)*	33 (66)

**Table 4:** Identification of dematiaceous species using various techniques (*N* = 50).

Identified Dematiaceous Species	Cases	PCR	KOH Examination	Calcofluor White-KOH Staining
*Alternaria alternata*	1	1	1	1
*Bipolaris hawaiiensis*	2	2	2	2
*Colletotrichum gloeosporioides*	2	2	2	2
*Curvularia lunata*	4	1	2	3
*Exophiala jeanselmei*	1	1	1	1
*Exserohilum rostratum*	2	2	2	2
*Fonsecaea pedrosoi*	1	1	1	1
*Lasiodiplodia theobromae*	6	6	5	6
*Microsphaeropsis arundinis*	1	1	1	1
*Papulaspora equi*	1	1	1	1
*Phaeoacremonium parasiticum*	1	1	1	1
*Phialophora verrucosa*	2	1	1	0
*Torula chromolaenae*	1	1	1	1
*Cladosporium* spp.^1^	4	0	1	1
*Graphium* spp.^2^	1	0	1	1
*Pleosporales* spp.^2^	1	1	1	1
Unidentified spp.	19	0	4	5
Summary	50	22	28	30

^1^*Cladosporium* spp. was identified by microscopic morphology only. ^2^ Only *Graphium* spp. and *Pleosporales* spp. could not be identified with the primer pairs used in this study. Abbreviations: PCR = polymerase chain reaction, spp. = species.

**Table 5:** Final treatment outcomes of dematiaceous fungal keratitis (*N* = 50).

Treatment Outcomes	Cases (%)
Medication success	24 (48%)
Medication failure	26 (52%)
- Therapeutic penetrating keratoplasty	17 (34%)
- Evisceration/enucleation	6 (12%)
- Scleral or corneal button patching	3 (6%)

Note: Treatment outcomes were defined as follows: medication success means no surgical intervention required, and medication failure means surgical interventions are mandatory.

**Table 6:** The comparison of demographics and clinical characteristics between the medication success and failure groups.

Parameters	Medication Success Group (*N* = 24)	Medical Failure Group (*N* = 26)	*p*-Value
Age (years) (mean, SD)	56.08, 11.66	54.69, 11.59	0.5797
Sex (male: female)	21:4	21:5	1.00
Diabetes mellitus (*N*, %)	4/24, 17%	5/26, 19%	0.9091
Underlying ocular disease (*N*, %)	5/24, 21%	6/26, 23%	1.00
- Previous globe rupture (*N*, %)	0/24, 0%	3/26, 11%	0.2625
Previous use of topical steroid (*N*, %)	5/24, 21%	6/26, 23%	1.00
Time to the presentation (days) (mean, SD)	9.96, 7.54	12.89, 15.78	0.1219
Visual acuity at presentation (LogMAR) (mean, SD)	1.33, 1.06	2.22, 0.68	**0.002**
Primary diagnosis as fungal keratitis (*N*, %)	7/24, 29%	8/26, 31%	0.7718
Trauma with foreign body exposure (*N*, %)	20/24, 83%	18/26, 69%	0.633
Area of infiltrate (mm^2^) (mean, SD)	12.84, 11.58	41.26, 41.79	**0.0021**
Depth (*N*, %)			**0.0072**
- One-third	4/24, 17%	1/26, 4%	
- Two-thirds	13/24, 54%	6/26, 23%	
- Full-thickness	7/24, 29%	19/26, 73%	
Perforation (*N*, %)	0/24, 0%	5/26, 19%	0.3816
Endophthalmitis (*N*, %)	0/24, 0%	2/26, 8%	0.5064
Hypopyon (*N*, %)	10/24, 42%	23/26, 88%	**0.0014**
Location of an ulcer (*N*, %)			**0.0025**
- Limbus	3/24, 12%	1/26, 4%	
- Central	15/24, 62%	6/26, 23%	
- Paracentral	6/24, 25%	11/26, 42%	
- Total	0/24, 0%	8/26, 31%	

Note: bold figures mean statistically significant.

**Table 7:** Clinical characteristics of dematiaceous fungal keratitis caused by *Cladosporium* spp., *Curvularia* spp., and *Lasiodiplodia* spp.

	*Cladosporium* spp. (*N* = 4)	*Curvularia* spp. (*N* = 4)	*Lasiodiplodia* spp. (*N* = 6)
Visual acuity at presentation (logMAR) (mean, SD)	2.43, 0.44	1.55, 1.17	2.01, 0.69
Exposure to an organic foreign body (*N*, %)	2/4, 50%	3/4, 75%	4/6, 67%
Time to the presentation (days) (mean, SD)	19.75, 7.59	4, 2.58	13, 9.51
Primary diagnosis with fungal keratitis (*N*, %)	0/4, 0%	1/4, 25%	1/6, 17%
Clinical characteristics (*N*, %)			
- Pigmentation	0/4, 0%	1/4, 25%	1/6, 17%
- Raised/elevated	0/4, 0%	3/4, 75%	1/6, 17%
- Epithelial plaque	0/4, 0%	2/4. 50%	1/6, 17%
- Feathery edge	1/4, 25%	2/4, 50%	5/6, 83%
- Hypopyon	4/4, 100%	2/4, 50%	4/6, 67%
Area of infiltrate (mm^2^) (mean, SD)	24, 23	27, 19	37, 46
Therapeutic penetrating keratoplasty or Corneal button patch (*N*, %)	0/4, 0%	2/4, 50%	5/6, 83%
Enucleation/evisceration (*N*, %)	2/4, 50%	0/4, 0%	0/6, 0%

## Supplementary Materials

The following are available online at https://www.mdpi.com/article/10.3390/jof7070526/s1. Sequenced data for species identification.
