# Supplementary material for: The Clinical Features and Prognostic Factors for Treatment Outcomes of Dematiaceous Fungal Keratitis over 9 Years at a Tertiary Eye Care in Northern Thailand
Source: J Fungi (Basel). 2021 Jun 30;7(7):526. doi: 10.3390/jof7070526 (PMC8303605; doi:10.3390/jof7070526)
Supplement: Supplementary file 1 [file jof-07-00526-s001.zip › jof-1263250-supplementary.pdf]

Supplementary result: Sequenced data for species identification

Alternaria alternata

CTGCGGAGGGATCATTACACAAATATGAAGGCGGGCTGGAACCTCTCGGGGTTACAGCCTTGCT
GAATTATTCACCCTTGTCTTTTGCGTACTTCTTGTTTCCTTGGTGGGTTCGCCCACCACTAGGACAA
ACATAAACCTTTTGTAATTGCAATCAGCGTCAGTAACAAATTAATAATTACAACTTTCAACAACG
GATCTCTTGGTTCTGGCATCGATGAAGAACGCAGCGAAATGCGATAAGTAGTGTGAATTGCAGA
ATTCAGTGAATCATCGAATCTTTGAACGCACATTGCGCCCTTTGGTATTCCAAAGGGCATGCCTGT
TCGAGCGTCATTTGTACCCTCAAGCTTTGCTTGGTGTTGGGCGTCTTGTCTCTAGCTTTGCTGGAGA
CTCGCCTTAAAGTAATTGGCAGCCGGCCTACTGGTTTCGGAGCGCAGCACAAGTCGCACTCTCTA
TCAGCAAAGGTCTAGCATCCATTAAGCCTTTTTTTCAACTTTTGACCTCGGATCAGGTAGGGATAC
CCGCTGAACTTAAGCATATCAATA

Bipolaris hawaiiensis

GATATGCTTAAGTTCAGCGGGTATCCCTACCTGATCCGAGGTCAAACGTGAGAAGGAGTCTTGAT
GGATTGCCGTCCTCTTTTGCTGATTGCAAGCGCAAAAATGTGCTGCGCTGCGAAACCAGTAGGCC
GGCTGCCAATCCTTTTAAGGCGAGTCCCCTTTTTGGGGGGGGACAAAAACGCCCAACACCAAGC
AAAGCTTGAGGGTACAAATGACGCTCGAACAGGCATGCCCTTTGGAATACCAAAGGGCGCAATG
TGCGTTCAAAGATTCGATGATTCACTGAATTCTGCAATTCACACTACGTATCGCATTTCGCTGCGT
TCTTCATCGATGCCAGAACCAAGAGATCCGTTGTTGAAAGTTGTAAATGATTTACATTTGTTATAC
TGACGCTGATTGCAACTGCATAAAAAAGGTTTATATTGTGGTCCTGGTGGCGAGCGAACCCGCCC
AGGAAACAACAAGTGCGCAAAAGACAAGGGTAATAAATAGTCCAGCCGCGAACGGCCTTCGTA
TTTTATTGTGTAATGATCCCTCCGCAG

Colletotrichum gloeosporioides **species complex**

ACCTGCGGAGGGATCATTACTGAGTTTACGCTCTATAACCCTTTGTGAACATACCTATAACTGTTG
CTTCGGCGGGTAGGGTCTCCGCGACCCTCCCGGCCTCCCGCCTCCGGGCGGGTCGGCGCCCGCCG
GAGGATAACCAAACTCTGATTTAACGACGTTTCTTCTGAGTGGTACAAGCAAATAATCAAAACTT
TTAACAACGGATCTCTTGGTTCTGGCATCGATGAAGAACGCAGCGAAATGCGATAAGTAATGTG
AATTGCAGAATTCAGTGAATCATCGAATCTTTGAACGCACATTGCGCCCGCCAGCATTCTGGCGG
GCATGCCTGTTCGAGCGTCATTTCAACCCTCAAGCTCTGCTTGGTGTTGGGGCCCTACAGCTGATG
TAGGCCCTCAAAGGTAGTGGCGGACCCTCCCGGAGCCTCCTTTGCGTAGTAACTTTACGTCTCGC
ACTGGGATCCGGAGGGACTCTTGCCGTAAAACCCCCCAATTTTCCAAAGGTTGACCTCGGATCAG
GTAGGAATACCCGCTGAACTTAAGCATATCA

Curvularia lunata

TCCTCCGCCTTATTGATATGCTTAAGTTCAGCGGGTATCCCTACCTGATCCGAGGTCAAACGTGAG
AAGGAGTCTTGATGGATTGCCGTCCTCTTTTGCTGATTGCAAGCGCAAAAATGTGCTGCGCTGCG
AAACCAGTAGGCCGGCTGCCAATCCTTTTAAGGCGAGTCTTTGGCAACCAAAGACAAAAAACGC
CCAACACCAAGCAAAGCTTGAGGGTACAAATGACGCTCGAACAGGCATGCCCTTTGGAATACCA
AAGGGCGCAATGTGCGTTCAAAGATTCGATGATTCACTGAATTCTGCAATTCACACTACGTATCG
CATTTCGCTGCGTTCTTCATCGATGCCAGAACCAAGAGATCCGTTGTTGAAAGTTGTAAATGATTT
ACATTTGTTATACTGACGCTGATTGCAACTGCATAAAAAAGGTTTATGATGTGGTCCTGGTGGCG
GGCGAACCCGCCCAGGAAACAACAAGTGCGCAAAAGACAAGGGTAATAAATAATTCAGCCTCC
GCAAAACTGGCCCGAGAGCACAGCCGCGTACAGCCTTCATATTTTATTGTGTAATGATCCCTCCG
CAGGTTCACCCTACGGA

Exophiala jeanselmei

TGCGGAGGGATCATTACCGAGTTAGGGTCCTCACAGGGCCCGACCTCCCAACCCTTTGTTTACGA
TACCTAGTGTTGCTTCGGTAGGCCTGGTCTCTGACCTGCCGGGGGGCCGTGAAACGCCCGCCGGA
GAGCGCCTGCCGACAGCCCCAACCTTTAAAATTCTTAACCAAACGTGACTTGTCTGAGTAAACGT
CTTTTAAATAAAAGCAAAACTTTCAACAACGGATCTCTTGGTTCTGGCATCGATGAAGAACGCAG
CGAAATGCGATAAGTAATGCGAATTGCAGAATTCTCGTGAGTCATCGAATCTTTGAACGCACATT
GCGCCCTTTGGTATTCCGAAGGGCATGCCTGTTCGAGCGTCATTTTCACCCCTCAAGCCCCGGCTT
GGTGTTGGACGGTTTGGTCTCGGGTCCGACCCCCCTTGACCCCTCCCAAAGACAATGACGGCGGG
CTGTTCGACCCCCGGTACACTGAGCATCTTAACGGAGCACGTACCGGTCTTTTGAGGGTCGACGG
CACCCGGTCTATACCTATATCTTTTTTTACAAGGTTGACCTCGGATCAGGTAGGAATACCCGCTGA
ACTTAAGCATATCA

Exserohilum rostratum

ACCTGCGGAGGGATCATTACTGAGTTTACGCTCTATAACCCTTTGTGAACATACCTATAACTGTTG
CTTCGGCGGGTAGGGTCTCCGCGACCCTCCCGGCCTCCCGCCTCCGGGCGGGTCGGCGCCCGCCG
GAGGATAACCAAACTCTGATTTAACGACGTTTCTTCTGAGTGGTACAAGCAAATAATCAAAACTT
TTAACAACGGATCTCTTGGTTCTGGCATCGATGAAGAACGCAGCGAAATGCGATAAGTAATGTG
AATTGCAGAATTCAGTGAATCATCGAATCTTTGAACGCACATTGCGCCCGCCAGCATTCTGGCGG
GCATGCCTGTTCGAGCGTCATTTCAACCCTCAAGCTCTGCTTGGTGTTGGGGCCCTACAGCTGATG
TAGGCCCTCAAAGGTAGTGGCGGACCCTCCCGGAGCCTCCTTTGCGTAGTAACTTTACGTCTCGC
ACTGGGATCCGGAGGGACTCTTGCCGTAAAACCCCCCAATTTTCCAAAGGTTGACCTCGGATCAG
GTAGGAATACCCGCTGAACTTAAGCATATCA

Fonsecaea pedrosoi

TTTCCTCCCGGCTTATGATATGCTTAAGTTCAGCGCGTATTCCTACCTGATCCGAGGTCAACCTTA
GAAAAAAAGTGTTCCCGTGAGGGAGAAGACCGGGTCCCGGGCGCCCTTCATCCGATACGTGCTC
AGTTAAGAAGCTCAGTGTACCGGGGGTTCCGCAGGCCGCCGTCATTGTCTTTAGGAGGGGTGGA
AAGTGTGAACTTACTCCACCAAGCCGTCCAACACCAAGCACAGGGGCTTGAGGGGGTGATAATG

ACGCTCGAACAGGCATGCCCTTCGGAATACCAAAGGGCGCAATGTGCGTTCAAAGATTCGATGA
CTCACTGGAATTCTGCAATTCGCATTACTTATCGCATTTCGCTGCGTTCTTCATCGATGCCAGAAC
CAAGAGATCCGTTGTTGAAAGTTTTTGCTTTTAATTGAATAAAATCACTTAGACATTGTAAATCAT
GATTGATCCAGAGTTAGGAGATTAGGCTATCGGCAGACACTGGCCAGAGGCAACGCCCGCATTG
AGCGGTCCTCCAGCGGTCAGCTTAAGACGGGCCTGCCGAAGCAACTGAGGTCAAGTAAACAAA
GGGTTGGGAGGTCGGGCCTAGAAGACCCTAACTCATTAATGATCCTTCCGCAGGTTA

Lasiodiplodia theobromae

CTTCCGTAGGTGAACCTGCGGAAGGATCATTACCGAGTTTTCGAGCTCCGGCTCGACTCTCCCAC
CCTTTGTGAACGTACCTCTGTTGCTTTGGCGGCTCCGGCCGCCAAAGGACCTTCAAACTCCAGTCA
GTAAACGCAGACGTCTGATAAACAAGTTAATAAACTAAAACTTTCAACAACGGATCTCTTGGTTC
TGGCATCGATGAAGAACGCAGCGAAATGCGATAAGTAATGTGAATTGCAGAATTCAGTGAATCA
TCGAATCTTTGAACGCACATTGCGCCCCTTGGTATTCCGGGGGGCATGCCTGTTCGAGCGTCATTA
CAACCCTCAAGCTCTGCTTGGAATTGGGCACCGTCCTCACTGCGGACGCGCCTCAAAGACCTCGG
CGGTGGCTGTTCAGCCCTCAAGCGTAGTAGAATACACCTCGCTTTGGAGCGGTTGGCGTCGCCCG
CCGGACGAACCTTCTGAACTTTTCTCAAGGTTGACCTCGGATCAGGTAGGGATACCCGCTGAACT
TAAGCATATCAATAAGCGGAGGAA

Papulaspora equi

CTGCGGAGGGATCATTACAGAGTTGCAAAACTCCCCAAACCATCGTGAACCTTTACCGCTAAAC
AGTTGCTTCGGCGGGTGGCCCGCGGGGGCCGCCGAGCCCGCCGGAGGAATCCAAAAAACCATC
GCTTTCAGTACCGGCCTCTCTGAGTTACTGTACTTTTAATTAAGTCAAAACTTTCAACAACGGATC
TCTTGGTTCTGGCATCGATGAAGAACGCAGCGAAATGCGATAAGTAATGTGAATTGCAGAATTC
AGTGAATCATCGAATCTTTGAACGCACATTGCGCCCGCCAGTATTCTGGCGGGCATGCCTGTTCG
AGCGTCATTTCCACCATCAGGCCCCCGGGCCTGTGTTGGGGACCTGCGGCTGCCCGCAGGCCCCG
AAATTCAGTGGCGGTCGCGCCGTAGCAACCGAGCGTAGTAACTAAAACGTCTCGCTCTGGCTCG
CTGGGCGCGCTCCGGCCGTAAAACACCCCCCCTTTTCGCAAAGGTTGACCTCGGATCAGGTAGG
AATACCCGCTGAACTTAAGCATATCAATAA

Phaeoacremonium parasiticum

TCCTCCGCTTATTGATATGCTTAAGTTCAGCGGGTATTCCTACCTGATCCGAGGTCAACCTTTGGA
AGTTGGGGTGTTTTACGGCTGGTAAAACCCACCAGGCGCTCCACAGCGAGAGAGAATTACTGCG
CTCGGAGTCCTGGCGAGCCCGCCACTGACTTTCGGGGCCCGCGCCCGCTGAGGGTGCGCGGCGC
CCCAACACCAGGCAACCAGGGCCTGAGGGTTGAAATGACGCTCGGACAGGCATGCCCGCCAGA
ATACTGGCGGGCGCAATGTGCGTTCAAAGATTCGATGATTCACTGAATTCTGCAATTCACATTAC
TTATCGCATTTCGCTGCGTTCTTCATCGATGCCAGAACCAAGAGATCCGTTGTTGAAAGTTTTTAC
TTGTTTGTAAAATATAACTCAGAGAGGTACGTTTTGTAATACAGAGTCTGTGCCCTCCGGCAGGC
CCACCCGGCCGGCCCCGCGAGGAGGCGGCCCGGCGGCGGCTTCAGGCCCGGAGGCCGTCCGCCT
TCACCTGCCGAAGCAACGAAAACAGGTATGTTCACAAAGGGTTTGGAGTACGAAACTCGTTAAT
GATCCCTCCGCAGGTTCACCCTACGGA

Phialophora verrucosa

TGCGGAAGGATCATTAACGAGTTAGGGTCTCTCCAGGCCCGACCTCCCAACCCTTTGTCTACCTT
GCTGCATGTTGCTTTGGCGGACCCGTCTCACGACCGCCCTGGGACCGCCGAGAGGCGTCCCTAGG
CCCGTGTCCGCCGATAGCCAACCTAAACTCTTGGATGAATCGTGTTTTATGTCTGAGTCTTGTGAT
TAAATCAAAAGCAAAAACTTTCAACAACGGATCTCTTGGTTCTGGCATCGATGAAGAACGCAGC
GAAATGCGATAAGTAATGCGAATTGCAGAATTCCCGTGAGTCATCGAATCTTTGAACGCACATTG
CGCCCTTTGGTATTCCAAAGGGCATGCCTGTTCGAGCGTCATTATCACCCCTCAAGCCCCGTGCTT
GGTGTTGGACGGATCTTGGTCGTGTGATCGCGACCCCTCCTAAAGACAATGACGGCGGCCTGTGG
ATCCCCCGGTACACTGAGCTTTTAACTGAGCACGTATCGGACAAGGGCGCCCAGGACCCGGTCC
TTCTCCTTTAACCAGGAAATTTCTTCAAGGTTGACCTCGGATCAGGTAGGAATACGCGCTGAACTT
AAGCATATCAAT

Torula chromolaenae

GAGGAAAAGAAACCAACAGGGATTGCCCCAGTAACGGCGAGTGAAGCGGCAACAGCTCAAATT
TGAAATCTGGCTCCTTTGGGGTCCGAGTTGTAATTTGCAGAGGGAGCTTTTGGCGTCGGTCGTGGT
CTAAGTTCCTTGGAACAGGACGTCACAGAGGGTGAGAATCCCGTACGTGGCCGCCGGCCTTCGC
CTTGTAAAGCCCCTTCGACGAGTCGAGTTGTTTGGGAATGCAGCTCTAAATGGGAGGTAAATTTC
TTCTAAAGCTAAATACCCGCCAGAGACCGATAGCGCACAAGTAGAGTGATCGAAAGATGAAAA
GCACTTTGGAAAGAGAGTGAAAAAGCACGTGAAATTGTTGAAAGGGAAGCGCTTGCAGCCAGA
CGTGCCCGCGGTTGATCATCCAGGCTCCAGCCTGGTGCACTCCTCCGCGGGCAGGCCAGCATCAG
TTCGGGCGGTCGGATAAAGGCCTCTATCACGTACCACCCCTCGGGGTGGCCTTATAGGGGAGGC
GCAACACGGCCAGCCCGGACTGAGGTTCCGCGCATCTGCTAGGATGCTGGCGTAATGGCGGCAA
GCGGCCCGTC

Pleosporales **spp.**

ACCTGCGGAAGGATCATTACTGTTGAAACGTCTCGGCCAGTCGTGTCCCGCATTGGGGCCTTCTG
CGCCAGCGTTTAGCACCCTTGATTTTGAGCACCTTTTGTTTCCTCGGCGGGTTCGCCCGCCAGCGA
GGACCCCCATGAACTCTTTGCAATAGCAATTTTTGTCTGAAATACAAACAATAATCAAAACTTTC
AACAATGGATCTCTTGGTTCTGGCATCGATGAAGAACGCAGCGAAATGCGATAAGTAGTGTGAA
TTGCAGAATTCAGTGAATCATCGAATCTTTGAACGCACATTGCGCCCCTCGGTATTCCGTGGGGC
ATGCCTGTTCGAGCGTCATTTAAACCCTCAAGCTCAGCTTGGTGTTGGGTGCTTGTCCCGCCTCCG
CGCGGCGACTCACCTCAAAGTCAGTGGCAGCCCGCATCAGCCGGCCGTGAGCGCAGCACAGTCG
CGCCCTAGGCCCCGGCAGGTCGGCTCTCCAGAAGCACATCTTCTTATTTTGACCTCGGATCAGGT
AGGGATACCCGCTGAACTTAAGCATATCAATA
